# Implementing enhanced recovery after bariatric surgery protocol: a retrospective study

**DOI:** 10.1007/s00540-015-2089-6

**Published:** 2015-10-24

**Authors:** Monika Proczko, Lukasz Kaska, Pawel Twardowski, Pieter Stepaniak

**Affiliations:** General Surgical Clinical Hospital for Endocrinology and Transplantology at the Gdansk University of Medicine in Poland, Gdansk, Poland; Department of Clinical Anesthesiology and Intensive Therapy at the Gdansk University of Medicine in Poland, Gdansk, Poland; Department of Operating Rooms, Catharina Hospital, P.O. Box 1350, 5602 ZA Eindhoven, The Netherlands

**Keywords:** Bariatric surgery, Early recovery after bariatric surgery, Gastric bypass, Sleeve gastrectomy and morbid obesity

## Abstract

While the demand for bariatric surgery is increasing, hospital capacity remains limited. The ERABS (Enhanced Recovery After Bariatric Surgery) protocol has been implemented in a number of bariatric centers. We retrospectively compared the operating room logistics and postoperative complications between pre-ERABS and ERABS periods in an academic hospital. The primary endpoint was the length of stay in hospital. The secondary endpoints were turnover times—the time required for preparing the operating room for the next case, induction time (from induction of anesthesia until a patient is ready for surgery), surgical time (duration of surgery), procedure time (duration of stay in the operating room), and the incidence of re-admissions, re-operations and complications during admission and within 30 days after surgery. Of a total of 374 patients, 228 and 146 received surgery following the pre-ERABS and ERABS protocols, respectively. The length of hospital stay was significantly shortened from 3.7 (95 % confidence interval [CI] 3.1–4.7) days to 2.1 (95 % CI 1.6–2.6) days (*P* < 0.001). Procedure (surgical) times were shortened by 15 (7) min and 12 (5) min for gastric bypass and gastric sleeve surgery, respectively (*P* < 0.001 for both), by introducing the ERABS protocol. Induction times were reduced from 15.2 (95 % CI 14.3–16.1) min to 12.5 (95 % CI 11.7–13.3) min (*P* < 0.001).Turnover times were shortened significantly from 38 (95 % CI 44–32) min to 11 (95 % CI 8–14) min. The incidence of re-operations, re-admissions and complications did not change.

## Introduction

Bariatric surgery is the only effective method of treating clinical obesity and the demand for this surgery is growing worldwide. If the demand for bariatric surgery increases as expected, while the hospital capacity remains unchanged, new strategies need to be found and implemented aimed at optimizing the peri- and postoperative care. A method for achieving this objective is the fast-track or enhanced recovery after surgery. This includes best practice preoperative preparation and standardization of procedures involved with peri- and postoperative care; all of which would ensure early recovery and improved prognosis [1–6]. Based on this principle, a number of bariatric centers have already implemented the Enhanced Recovery After Bariatric Surgery (ERABS) protocol. We retrospectively compared the operating room logistics and postoperative complications between patients receiving surgery following the pre-ERABS (2013–2014) and ERABS protocol (January to June 2015) in an academic hospital. During both periods, patients underwent one of the following laparoscopic surgeries—Roux-en-Y gastric bypass, sleeve gastrectomy, gastric banding or revisional surgery—in a specialized operating room (Karl Storz OR1™). The primary endpoint was the length of stay in hospital and was measured in days, i.e., the day the patient was admitted until discharge. Secondary endpoints were turnover times (the time required for preparing the operating room for the next case), induction time (from induction of anesthesia until a patient is ready for surgery), surgical time (duration of surgery), procedure time (duration of stay in the operating room), the number of re-admissions, re-operations and complications during admission and within 30 days. Complications were graded using the Clavien−Dindo classification [7, 8]. The patients were initially qualified for the procedure during their first visit to the clinical surgical advice center, based on International Federation for the Surgery of Obesity (IFSO) criteria.

## Statistics

Differences between the pre-ERABS and ERABS period in continuous data were analyzed using unpaired Student *t* test, and differences in categorical data were analyzed using the chi-squared test. A *p* value <0.05 was considered statistically significant.

### The pre-ERABS protocol

As part of an operating room (OR) schedule, two to three procedures were performed in a day by a team of surgeons, anesthesiologist and OR nurses in one specialized OR (Karl Storz OR1™). Patients received paracetamol 1 g and diazepam 5 mg orally as premedication 1 h before surgery. Pneumatic compression stockings were adjusted and low-molecular-weight heparin 5,000 IU was given subcutaneously for thrombosis prophylaxis. Patients were transported to the OR on the bed and then moved to the operating table. General anesthesia was induced with propofol, sufentanil and rocuronium with the dose adjusted to corrected body weight. After tracheal intubation, anesthesia was maintained with sevoflurane or propofol with intermittent administration of sufentanil to maintain the bispectral index between 40 and 60. Rocuronium was continuously infused with monitoring of the train-of-four (TOF) ratio. Neuromuscular blockade was reversed with atropine and neostigmine on completion of surgery. Administration of anesthetics was discontinued when the TOF ratio recovered to >90 % and the endotracheal tube removed. Patients were transported to the post-anesthesia care unit (PACU), where patient-controlled analgesia was provided using morphine. Patients were immobilized by compression stockings, urinary catheters and opiates for analgesia and were highly dependent on the nursing staff.

### The ERABS protocol

Five to six procedures were performed in a day by a dedicated team consisting of fixed surgeons, a dedicated anesthesiologist and OR nurses [9, 10].

## Anesthesia

Patients received low-molecular-weight heparin 5,000 IU subcutaneously on the evening before surgery. Without premedication, patients without compression stockings walked to the operating room, and then receive a balanced crystalloid solution via a peripheral venous catheter. General anesthesia was induced with sufentanil 10 µg and remifentanil 0.5 µg/kg, followed by continuous infusion at 0.1 µg/kg/min, with propofol 1.5–2 mg/kg and rocuronium 0.6 mg/kg before orotracheal intubation. Doses of these anesthetics were decided based on ideal body weight. The lungs were ventilated to maintain end-tidal carbon dioxide tension between 32 and 35 mmHg with a positive end-expiratory pressure of 3–10 cmH_2_O. Anesthesia was maintained with desflurane 0.8–1.0 × minimum alveolar concentration, with a mixture of air and oxygen with an oxygen concentration of 50–60 % to maintain the bispectral index between 40 and 60. At the beginning of surgery, dexamethasone 8 mg and metamizole 2.5 g were administered as an antiemetic and analgesic, respectively. Following anastomosis of the intestinal organs, ondansetron 8 mg and oxycodone 10 mg were administered intravenously.

A maximum of 1,000 ml of balanced crystalloid solution was administered during surgery. Both desflurane and remifentanil were discontinued at a pre-defined point according to the type of surgery. On completion of surgery, a combination of atropine and neostigmine, or sugammadex alone was administered for reversal of neuromuscular blockade. The endotracheal tube was removed after confirming the TOF ratio >90 %, SpO_2_ >92 %, PaO_2_ >60 mmHg, adequate ventilation, stable blood pressure and heart rate, usually within 5 min after surgery. The patients were moved to their bed unaided by medical personnel and were transferred to the PACU.

## After anesthesia

On the PACU, patients received dexamethasone 8 mg and ondansetron 8 mg. The level of pain was evaluated using a visual analog scale. Patients who leave the PACU are transported to the surgical ward after confirming PACU discharge criteria—(1) respond to verbal stimuli, and (2) respiratory rate 10–20/min, SpO2 >95 % (at room air), and heart rate 60–90 bpm. Patients are encouraged to leave their bed and move around independently by the nurses to minimize the risk of venous thrombosis, and are discharged as soon as satisfying the following conditions, without complications during anesthesia and surgery—(1) heart rate <100 bpm, (2) respiratory rate <20/min, (3) body temperature <38 °C, (4) VAS <4/10 with acetaminophen 4 g daily, (5) recovery of full mobility, being able to independently perform basic activities, and (6) C-reactive protein <100 mg/l or decrease compared with the first postoperative measurements and the number of leucocytes <20,000/µl. On the surgical ward, patients were administered a daily dose of acetaminophen 4 g or tramadol 150 mg, and additional paracetamol 150 mg or diclofenac 150 mg followed by dexamethasone 8 mg, metoclopramide 30 mg, ondansetron 12 mg and pantoprazole 80 mg. The patients were required to visit our hospital 1, 4 and 12 months after discharge for follow-up.

A total of 374 patients were included in this study—228 underwent surgery following the pre-ERABS protocol and 146 following the ERABS protocol. Of the 374 patients, 186, 144, 31 and 13 underwent gastric bypass, sleeve gastrectomy, gastric band and revisional surgery, respectively. There were no differences in baseline demographic or co-morbidity data between the two groups of patients (Table 1). The length of stay in the hospital was significantly shortened from 3.7 (95 % CI 3.1–4.7) days to 2.1 (95 % CI 1.6–2.6) days (*p* < 0.001) following the ERABS protocol compared to the pre-ERABS protocol. Procedure (surgical) times were shortened by 15 (7) min and 12 (5) min for gastric bypass and gastric sleeve surgery, respectively (*P* < 0.001 for both), by introducing the ERABS protocol. Induction times were reduced from 15.2 (95 % CI 14.3–16.1) min to 12.5 (95 % CI 11.7–13.3) min (*P* < 0.001). Turnover times shortened significantly from 38 (95 % CI 44–32) min to 11 (95 % CI 8–14) min. The incidence of re-operations, readmissions and complications within 30 days after discharge did not change after implementation of the ERABS protocol (Table 2). The reduction in the length of stay in hospital can be explained by the change in the anesthesia protocol; this ensured that the patient became mobile as soon as possible following the surgical procedure, quickly regaining full mobility, including oral food intake. The procedure time decreased by shortening the induction time and surgical time but also as a result of introducing the ‘working with a fixed team’ concept. A dedicated group of surgeons, anesthesiologist and circulating nurses worked together. Instead of operating on two to four patients per day on several days of the week with different anesthesiologist and nurses, the team now works together all day for 2 days per week. This contributes to team cohesion and may increase the team work and safety climate and productivity [9, 10]. In conclusion, our study confirms that the use of ERABS makes it possible to create centers which are able to process a large number of bariatric cases and at the same time maintain the quality as well as efficiency of surgery, while ensuring the highest safety standards [11, 12].

**Table 1:** Baseline demographic and co-morbidity data

	Pre-ERABS	ERABS	*P* value
No. of patients	228	146	
Age (years)	44 ± 10	43 ± 11	0.365^a^
Length of stay in hospital (days)	3.7 ± 0.4	2.1 ± 0.5	<0.001
No. of male patients	141 (62 %)	85 (58 %)	0.485^b^
Body mass index (kg/m^2^)	45.1 ± 4.6	44.4 ± 4.7	0.154^a^
Visual analog score	2.6 ± 1.4	2.4 ± 1.1	0.145^a^
Medical co-morbidity, no. (%)^b^			
Hypertension	91 (39.8 %)	65 (44.5 %)	0.388
Type 2 diabetes mellitus	59 (25.9 %)	51 (34.9 %)	0.061
Obstructive sleep apnea	37 (16.2 %)	22 (15.1 %)	0.764
Current smoker	68 (29.8 %)	45 (30.8 %)	0.838
COPD	22 (9.6 %)	16 (11.7 %)	0.683
GERD	46 (20.1 %)	24 (16.4 %)	0.366
Dyslipidemia	34 (14.9 %)	13 (8.9 %)	0.087
Primary procedures^b^			
Gastric bypass	113 (49.6 %)	73 (50 %)	0.934
Sleeve gastrectomy	89 (39.0 %)	55 (37.7 %)	0.791
Gastric band	18 (7.9 %)	13 (8.9 %)	0.730
Revisional	8 (3.5 %)	5 (3.4 %)	0.965

Values are expressed as mean ± standard deviation or absolute value (%)

*COPD* chronic obstructive pulmonary disease, *GERD* gastroesophageal reflux disease

^a^Using independent *t* tests

^b^Using chi-squared test

**Table 2:** Complications, re-admissions and re-operations

Within 30 days after discharge			
	Pre-ERABS	ERABS	*P* value^a^
No. of patients	228	146	
Clavien−Dindo classification complications^b^			
Minor	20 (8.77 %)	15 (10.27 %)	0.626
Grade I	12 (5.26 %)	8 (5.48 %)	0.928
Grade II	8 (3.51 %)	7 (4.79 %)	0.672
Major	6 (2.63 %)	4 (2.74 %)	0.958
Grade IIIa	2 (0.88 %)	1 (0.68 %)	0.834
Grade IIIb	2 (0.88 %)	2 (1.37 %)	0.656
Grade IVa	1 (0.44 %)	1 (0.68 %)	0.754
Grade IVb	1 (0.44 %)	0 (0.00 %)	0.421
Re-admissions	8 (3.51 %)	4 (2.74 %)	0.672
Re-operations	3 (1.32 %)	2 (1.37 %)	0.971
Mortality	1 (0.44 %)	0 (0.00 %)	0.421

^a^Using chi-squared test

^b^ Grade I: any deviation from the normal postoperative course without the need for pharmacological treatment or surgical, endoscopic and radiological interventions. Acceptable therapeutic regimens are drugs as anti-emetics, antipyretics, analgetics, diuretics and electrolytes and physiotherapy. This grade also includes wound infections opened at the bedside. Grade II: requiring pharmacological treatment with drugs other than such allowed for grade I complications. Blood transfusions and total parenteral nutrition are also included. Grade III: requiring surgical, endoscopic or radiological intervention. Grade III-a: intervention not under general anesthesia. Grade III-b: intervention under general anesthesia. Grade IV: life-threatening complication requiring IC/ICU management. Grade IV-a: single organ dysfunction (including dialysis). Grade IV-b: multi organ dysfunction. Grade V: death of a patient [7.8]
